# Ultrasound-guided stellate ganglion block benefits the postoperative recovery of patients undergoing laparoscopic colorectal surgery: a single-center, double-blinded, randomized controlled clinical trial

**DOI:** 10.1186/s12871-024-02518-5

**Published:** 2024-04-10

**Authors:** Di-han Lu, Xuan-xian Xu, Rui Zhou, Chen Wang, Liang-tian Lan, Xiao-yu Yang, Xia Feng

**Affiliations:** 1 Department of Anesthesiology, The First Affiliated Hospital, Sun Yat-Sen University, No.58 2nd Zhongshan Road, Guangzhou, Guangdong 510080 P.R. China; 2 Department of Hepatobiliary Surgery, The Sun Yat-Sen Memorial Hospital, Sun Yat-Sen University, No. 107 Yanjiang West Road, Guangzhou, Guangdong 510120 P.R. China

**Keywords:** Stellate ganglion block, Colorectal surgery, ERAS

## Abstract

**Background:**

With the increasing prevalence of colorectal cancer (CRC), optimizing perioperative management is of paramount importance. This study investigates the potential of stellate ganglion block (SGB), known for its stress response-mediating effects, in improving postoperative recovery. We postulate that preoperative SGB may enhance the postoperative recovery of patients undergoing laparoscopic CRC surgery.

**Methods:**

We conducted a randomized controlled trial of 57 patients undergoing laparoscopic colorectal cancer surgery at a single center. Patients, aged 18–70 years, were randomly assigned to receive either preoperative SGB or standard care. SGB group patients received 10 mL of 0.2% ropivacaine under ultrasound guidance prior to surgery. Primary outcome was time to flatus, with secondary outcomes encompassing time to defecation, lying in bed time, visual analog scale (VAS) pain score, hospital stays, patient costs, intraoperative and postoperative complications, and 3-year mortality. A per-protocol analysis was used.

**Results:**

Twenty-nine patients in the SGB group and 28 patients in the control group were analyzed. The SGB group exhibited a significantly shorter time to flatus (mean [SD] hour, 20.52 [9.18] vs. 27.93 [11.69]; *p* = 0.012), accompanied by decreased plasma cortisol levels (mean [SD], postoperatively, 4.01 [3.42] vs 7.75 [3.13], *p* = 0.02). Notably, postoperative pain was effectively managed, evident by lower VAS scores at 6 h post-surgery in SGB-treated patients (mean [SD], 4.70 [0.91] vs 5.35 [1.32]; *p* = 0.040). Furthermore, patients in the SGB group experienced reduced hospital stay length (mean [SD], day, 6.61 [1.57] vs 8.72 [5.13], *p* = 0.042).

**Conclusions:**

Preoperative SGB emerges as a promising approach to enhance the postoperative recovery of patients undergoing laparoscopic CRC surgery.

**Clinical trial registration:**

ChiCTR1900028404, Principal investigator: Xia Feng, Date of registration: 12/20/2019.

## Introduction

Colorectal cancer (CRC) ranks as the fourth most commonly diagnosed and third deadliest cancer globally [1, 2]. Currently, radical tumor resection, coupled with appropriate chemotherapy, remains the most efficacious treatment [3]. Although minimally invasive techniques have accelerated the postoperative recovery of colorectal surgery patients, the management of CRC surgeries still entails substantial challenges [4]. Notably, these challenges encompass extensive operative wounds, acute postoperative pain, considerable stress responses, and a spectrum of complications that extend hospital stays and escalate treatment expenses. Thus, optimizing perioperative management stands as a pivotal concern in the field of perioperative medicine.

In recent years, the concept of enhanced recovery after surgery (ERAS) has gained substantial traction across various surgical disciplines, particularly in colorectal surgeries [5]. Anchored in pain management, early mobilization, and perioperative stimulation of intestinal function, ERAS aims to curtail the recovery phase [6]. Given the potent impact of the activated sympathetic nervous system (SNS) in the postoperative period, strategies to mitigate the stress response assume significance in optimizing recovery [7]. However, contemporary methods to quell the SNS, encompassing sedatives, analgesics, and psychological interventions, yield limited effectiveness and entail undesirable side effects.

Stellate ganglion block (SGB) has exhibited promise in fostering postoperative intestinal function recovery [8–10], concurrently tempering stress responses [10]. However, the potential benefits of SGB in facilitating rapid perioperative rehabilitation remain to be fully understood. Particularly, there is a lack of research on the recovery outcomes other than stress or intestinal indicators. Furthermore, advancements in ultrasound-guided techniques have rendered SGB a safe and efficacious intervention [11, 12]. Thus, this randomized controlled trial aims to assess the impact of preoperative ultrasound-guided SGB on postoperative recovery, complications, stress responses, and treatment costs in CRC surgery patients. The primary outcome of this study is the time to flatus, while secondary outcomes encompass time to defecation, duration of bed rest, pain intensity, hospitalization duration, patient costs, intraoperative and postoperative morbidity, as well as 3-year mortality.

## Materials and methods

### Trial design and oversight

**Fig. 1:**
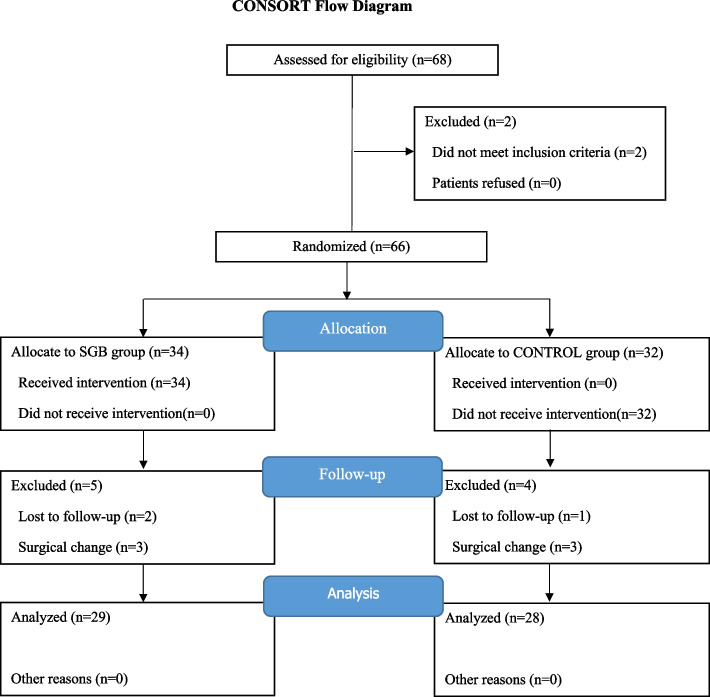
CONSORT flow chart for this study. CONSORT indicates consolidated standards of reporting trials

The prospective, randomized, double-blinded, single-center trial was approved by the Ethics Committee of the First Affiliated Hospital of Sun Yat-sen University (Guangzhou, Guangdong, China. No. [2019]334, chairperson Churong Ji) on 12/09/2019. Written informed consent was obtained from all subjects participating in the trial. The trial was registered prior to patient enrollment at Chinese Clinical Trial Registry (ChiCTR1900028404, Principal investigator: Xia Feng, Date of registration: 12/20/2019). At enrollment, all participants provided written informed consent. The Consolidated Standards of Reporting Trials (CONSORT) guidelines were followed in this study (Fig. 1). Recruitment was discontinued when enrollment reached the number suggested by projections to yield sufficient statistical power. A research monitor, independent of the investigative team and approved by Human Research Protection Office, served as an advocate for the safety of the study participants. The research monitor thoroughly reviewed all amendments to the protocol as well as any adverse events, protocol deviations, and other relevant event reports. The monitor diligently assessed the accumulating data from a clinical trial in terms of progress, participant safety, critical efficacy results, and subsequently offer recommendations for potential modifications, continuations or terminations if necessary.

### Trial participants

Key inclusion criteria included aged 18-70 yr, with the American Society of Anesthesiology (ASA) I-III undergoing laparoscopic radical resection of CRC, fit for elective surgery, and a preoperatively signed informed consent. Key exclusion criteria included emergency operations, operation with enterostomy, patients with communication difficulties before surgery, other serious underlying diseases, and brady-arrhythmias.

All patients were asked to fast preoperatively without premedication. Standard monitoring, including non-invasive blood pressure, heart rate, continuous ECG, oxygen saturation, and end-tidal carbon dioxide, was performed before anesthesia induction. Intravenous access was established, and patients were kept warm with a thermal blanket upon entering the room. Nasopharyngeal temperature, central venous pressure (CVP), Narcotrend value were also recorded after anesthesia induction during the surgery. General anesthesia was administered to all patients, as described below.

Participants were randomized by random numbers generated by software to either a normal saline group (Control group) or a ropivacaine group (SGB group) preoperatively. Software (Microsoft Excel, Version 2013, USA, www.microsoft.com) was used for randomization. Following allocation results, the medication was prepared by an anesthesiologist who was not involved in the study. Study medication included either 10 mL of normal saline (Control group) or 10 mL of 0.2% ropivacaine (SGB group). Anesthesiologists and surgeons were blinded to different local anesthetic regimes.

### Anesthesia procedures and perioperative management

All patients enrolled in this trial received general anesthesia. Anesthesia induction involved intravenous administration of propofol (2 mg/kg), sufentanil (0.2 ug/kg) and cisatracurium (0.2 mg/kg). Following tracheal intubation, propofol (TCI, 1-2ug/ml), remifentanil (TCI, 2–4 ng/ml), and 1% sevoflurane were administered to maintain anesthesia. Internal jugular vein puncture and catheterization were performed after the SGB procedure. Narcotrend monitoring was employed to regulate the sedative stage, maintaining values within the range of 40–60. Perioperative analgesic and anti-emetic management were standardized as sufentanial and palonosetron. Patient-controlled intravenous analgesia (PCVA) with sufentanil was used for postoperative analgesia. The PCVA regimen typically involved a mixture of sufentanil (150 μg) with normal saline, resulting in a total volume of 150 ml. Sufentanil was administered via a pump programmed to deliver a continuous background infusion at a rate of 1 ml/h, with an additional 2 ml available on demand. Patients with a postoperative VAS score of 4 or higher were permitted to self-administer the necessary bolus dose by pressing a button until their VAS score reached ≤ 3.

### Ultrasound-guided SGB procedure

**Fig. 2:**
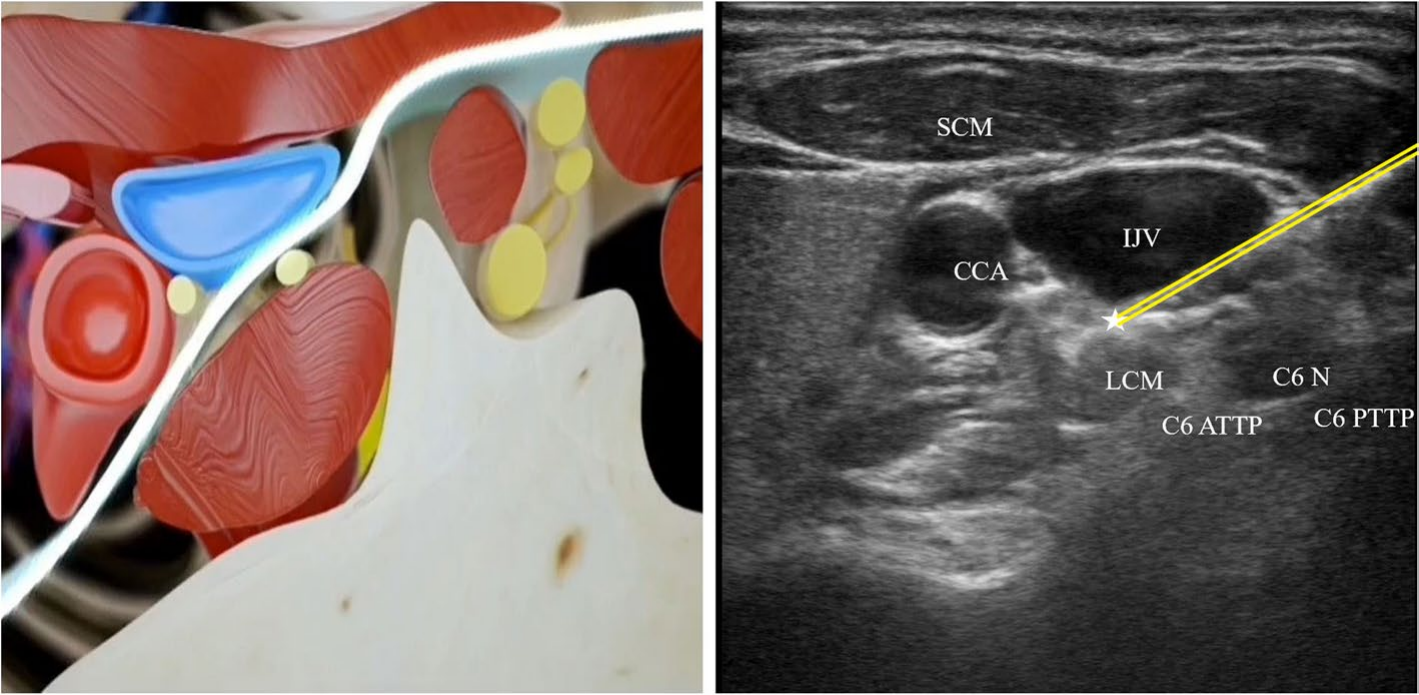
Schematic diagram of the SGB procedure. PTTP, posterior tubercle of transverse process. ATTP, anterior tubercle transverse process. N, nerve. LCM, longus colli musculus. IJV, internal jugular vein. CCA, common carotid artery. SCM, sternocleidomastoid muscle. ☆, target site

The ultrasound-guided SGB was performed after induction of anesthesia. Patients were in the supine position for the ultrasound-guided (X-PORTE; SonoSite Inc., USA) SGB, which was performed by experienced anesthesiologists (who has performed ultrasound-guided SGB for over 30 cases) to identify appropriate anatomical landmarks, avoid intravascular injection, and guide injectate placement [13]. 10 mL of ropivacaine, 0.2%, was injected around and into the site of the ganglion at the level of the C6 anterior tubercle after a negative puff test to exclude intravascular injection and a negative cerebrospinal fluid aspiration. Under out-of-plane ultrasound guidance, a 20-gauge Tuohy needle was applied percutaneously to the anterior or anterolateral edge of the longus colli muscle for participants receiving the active SGB. A short-axis ultrasound view confirmed injectate spread along the longus colli muscle (Fig. 2). Efficacy of the block was confirmed by the specific observer who is aware of the assignment by a temperature change of at least 1 °C in the ipsilateral upper extremity. Horner sign was not included for the patients has already intubated after induction of anesthesia. The sham procedure used the same technique, except normal saline was injected in soft tissues superficial to the anterior tubercle of C6 [14]. All other clinical and study personnel were unaware of treatment assignment.

Possible adverse events related to SGB and their preventive and treatment measures are as follows: potential severe complications, such as intravascular injections, retropharyngeal hematoma, and brachial plexus injury, may arise [15]. While ultrasound guidance was utilized, absolute prevention of these events cannot be guaranteed. Therefore, preventive measures include: precise identification of tissue fascia planes and crucial blood vessels via pre-scan ultrasound, careful planning of a safe needle insertion trajectory, and immediate cessation of the procedure upon encountering a blood vessel or important structure, followed by appropriate handling.

Treatment measures involve applying adequate compression to manage bleeding upon vessel contact, vigilant monitoring of vital signs for signs of local anesthetic poisoning (e.g., increased heart rate), continuous intraoperative ultrasound surveillance for progressive changes in the affected area, post-surgical assessment of nerve damage, swallowing, and vocal function changes, and timely involvement of otolaryngology and vascular surgery departments for assistance as needed.

### Outcome measurements

Outcome analysis was conducted via per-protocol analysis. The primary outcome was the time to flatus after surgery as previously reported [9, 10]. As secondary endpoints, we measured the time to the first bowel movement, serum cortisol levels, a solid diet tolerance, additional analgesic requirements, intraoperative complications, postoperative pain score, postoperative complications, hospital stay duration, and 3-year mortality.

The time to flatus was defined as the time to the first audible bowel sound during routine postoperative care. The nurses or assistants checked the patients hourly for bowel sounds and asked them to note the time to flatus and defecation and then inform them. Pain was assessed using a VAS ranging from 0 (no pain) to 10 (extreme pain). The pain assessment was carried out by an assistant doctor who was unaware of the clinical trial, as part of the clinical care given to all patients (6, 12, 24, and 48 h postoperatively). The hospital stay was defined as the number of nights spent in the hospital after surgery. The period of tolerating a solid diet was measured from the time the patient awoke from anesthesia until the time they were able to consume solid food (i.e., any food requiring chewing) without vomiting or experiencing significant nausea within 4 h of consuming the meal. Lying in bed time was defined as the time from returning to the bed in the ward after surgery until the first ambulation. Intraoperative complication includes, but not limited to, hypotension, cardiovascular event, severe subcutaneous emphysema, etc. Postoperative complication includes, but not limited to, nausea, vomiting, abdominal distension, hypotension, dizziness, fever and ileus, etc. A sample of 5 mL of peripheral blood was collected before induction of anesthesia and 0.5 h after surgery. We centrifuged the blood specimen at 3000 rpm for 5 min, and the supernatant was stored in the refrigerator at -80 °C. Enzyme linked immunosorbent assay (ELISA) was used to measure the stress hormone cortisol in the serum, a biomarker of stress responses. Blood glucose levels were also measured preoperatively (just as anesthesia begins) and postoperatively (30 min post operation). Additionally, analgesic requirements were assessed using electronic records, and the total consumption of sufentanil was calculated. In addition, we assessed postoperative emotional states of patients using the Hamilton Anxiety Scale (HAMA) [16] and the Patient Health Questionnaire-9 (PHQ-9) [17] after surgery on the third postoperative days. All patients were followed for at least 3 years to obtain the mortality rates.

### Detection of serum cortisol by ELISA

The detection of serum cortisol by ELISA has been described before [18]. Before determination, the serum supernatant was reconstituted in 4 °C ice water, then centrifuged again at 3000 rpm for 5 min. The corticosterone levels were determined by using an ELISA kit (Abbott, U.S.) according to the manufacturer's instructions. The procedure was strictly adhered to, and the absorbance (OD values) of each well was determined at 450 nm wavelength. The multinomial quadratic regression equation of the standard curve was calculated using the concentration of the standard material as the longitudinal coordinate and the OD value as the transverse coordinate. The OD value of the sample was replaced by the equation, and the concentration of the sample was calculated, multiplied by the dilution multiple, which represents the actual concentration.

### Statistical analysis

The average time to flatus of CRC patients at our center was calculated using our preliminary experimental results. Based on our pilot findings, the average time to flatus was about 27 h in the control group versus about 20 h in the SGB group as more than 25% reduction in time after SGB procedure. On the basis of our institutional data, a sample size of at least 31 patients per arm was calculated to have a power of 0.80 and a significance level of 0.05. Finally, we included 34 patients in each group to account for possible dropouts. Variables were reflected as means with standard error of mean or interquartile range. In the case of continuous variables, t-tests were used when they were normally distributed, otherwise, Mann–Whitney U tests were used. The χ2 test was used for comparison of categorical variables and 2-way ANOVA for repeated measures was performed for comparisons between the groups in time. Nonparametric data were analyzed using Kruskal–Wallis tests. The statistical analyses were performed using GraphPad Prism version 8.0 (GraphPad Software, San Diego, California). *P* < 0.05 were considered significant.

## Results

### Participants

A total of 68 individuals were prescreened to determine basic eligibility, and 66 individuals (97.1%; 41 men and 25 women; mean [SD] age, 57.37 [11.83] years) were subsequently randomized to receive treatment (34 to SGB and 32 to sham). Of these, 57 individuals (86.4%) completed the study through the follow-up period (Fig. 1).

### Baseline characteristics

Baseline demographic and clinical characteristics of study participants were comparable between the two groups (Table 1). Patients who completed the study are detailed in Table 1. No statistically significant differences were observed in age, gender, BMI, ASA scale, operation time, or anesthesia time. In particular, intraoperative usage of sufentanil (median [interquartile range], SGB group, 35 [9]; Control group, 35 [4]) did not significantly differ (Table 1).

**Table 1:** Demographic and surgical profiles of the patients

Characteristic	SGB group(*n* = 28)	Control group(*n* = 29)	*p*
Age (yr ± SD)	57.47 ± 12.68	56.28 ± 12.20	.716^*^
Sex(%)			.308^‡^
Male, n(%)	16(58.6)	21(72.4)	
Female, n(%)	12(41.4)	8(27.6)	
BMI (kg/m^2^, mean ± SD)	22.51 ± 3.17	23.03 ± 3.15	.534^*^
ASA I/II	11/16	14/15	.503^‡^
Duration of anesthesia, min	293.79 ± 105.11	274.33 ± 82.86	.840^†^
Duration of surgery, min	237.93 ± 100.30	221.59 ± 68.10	.882^†^
Intraoperative sufentanil, ug			.265^†^
Medium	35	35	
Interquartile Range	9	5	

^*^:student test

^†^:Mann–Whitney test

^‡^:χ2 test

### SGB significantly shortened time to flatus postoperative

**Fig. 3:**
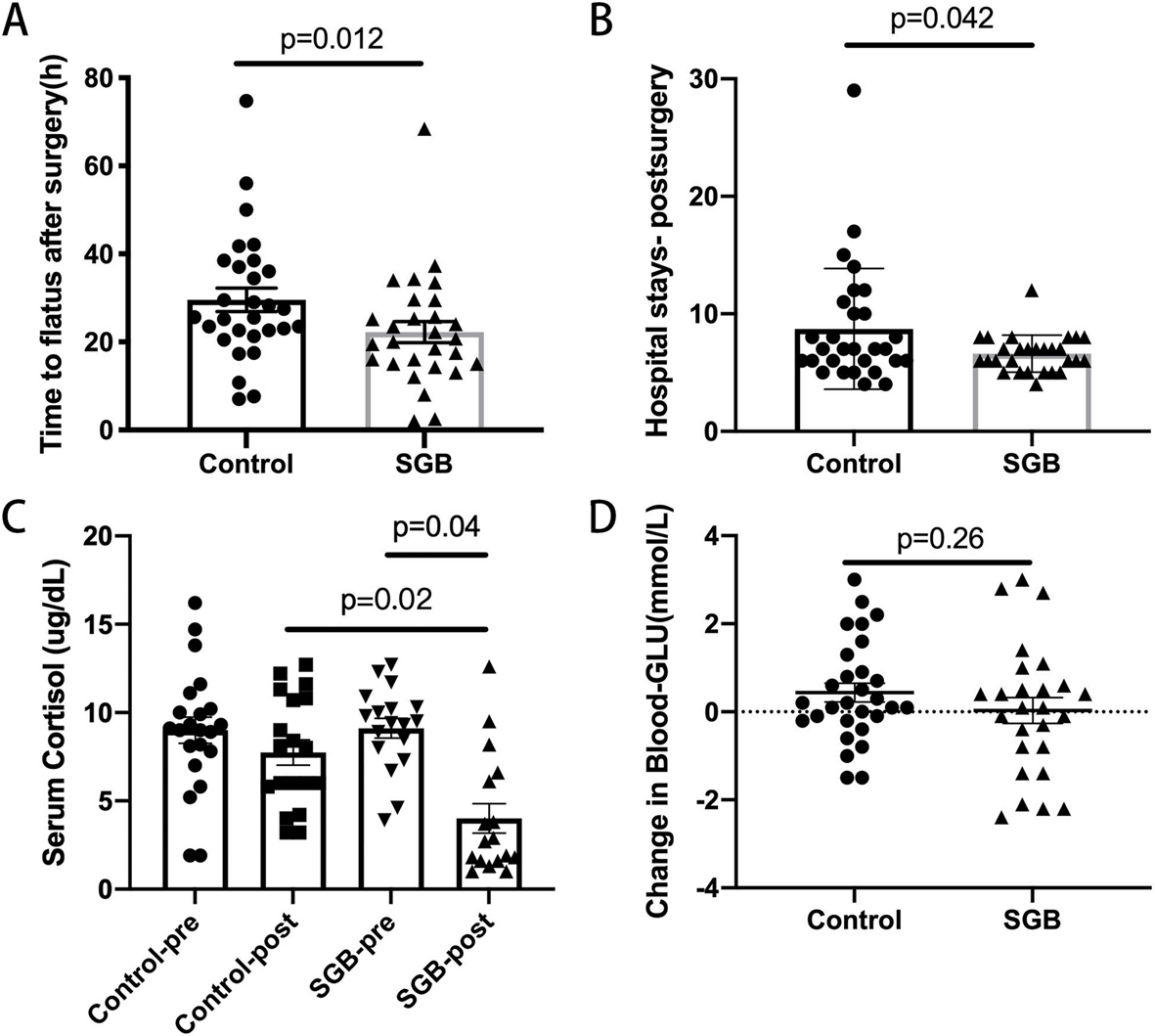
Typical postoperative outcomes, time to flatus after surgery (**A**), length of hospital stays (**B**), serum cortisol (**C**) and glucose (**D**) levels between Control group and SGB group. Data are presented as mean ± SD. SGB, stellate ganglion block. *P* values are presented

The primary outcome, assessed for all included patients (Table 2), revealed that the time to first flatus, a key trial parameter, was significantly shorter in the SGB group compared to the control group (mean [SD] hour, 20.52 [9.18] vs. 27.93 [11.69]; *p* = 0.012; Table 2, Fig. 3A). However, the time to defecation (mean [SD] hour, 33.14 [17.83] vs. 38.97 [22.48]; *p* = 0.369) and lying in bed time (mean [SD] hour, 38.85 [16.82] vs. 36.85 [16.08]; *p* = 0.687) did not differ significantly between the groups (Table 2).

**Table 2:** Postoperative outcomes

Characteristic	SGB group(*n* = 28)	Control group(*n* = 29)	*p*
Time to flatus, h	20.52 ± 9.18	27.93 ± 11.69	.012^*#^
Time to defecation, h	33.14 ± 17.83	38.97 ± 22.48	.369^*^
Lying in bed time, h	38.85 ± 16.82	36.85 ± 16.08	.687^*^
Hospital stays, d	12.39 ± 3.44	15.79 ± 6.64	.019^*#^
Hospital stays post-surgery, d	6.61 ± 1.57	8.72 ± 5.13	.042^*#^
Patient costs, RMB	94,484.78 ± 20,545.96	95,135.39 ± 21,514.85	.910^*^
Intraoperative morbidity(N/Y)	17/11	20/9	.514^‡^
Postoperative morbidity(N/Y)	26/2	21/8	**.042**^**‡#**^
3-year mortality(N/Y)	1/25	2/24	.999^‡^

^#^*P* < 0.05 indicates statistically significant values

^*^:student test

^‡^:χ2 test

### Reduced VAS score and plasma cortisol levels 6 h after surgery in patients of SGB group

**Fig. 4:**
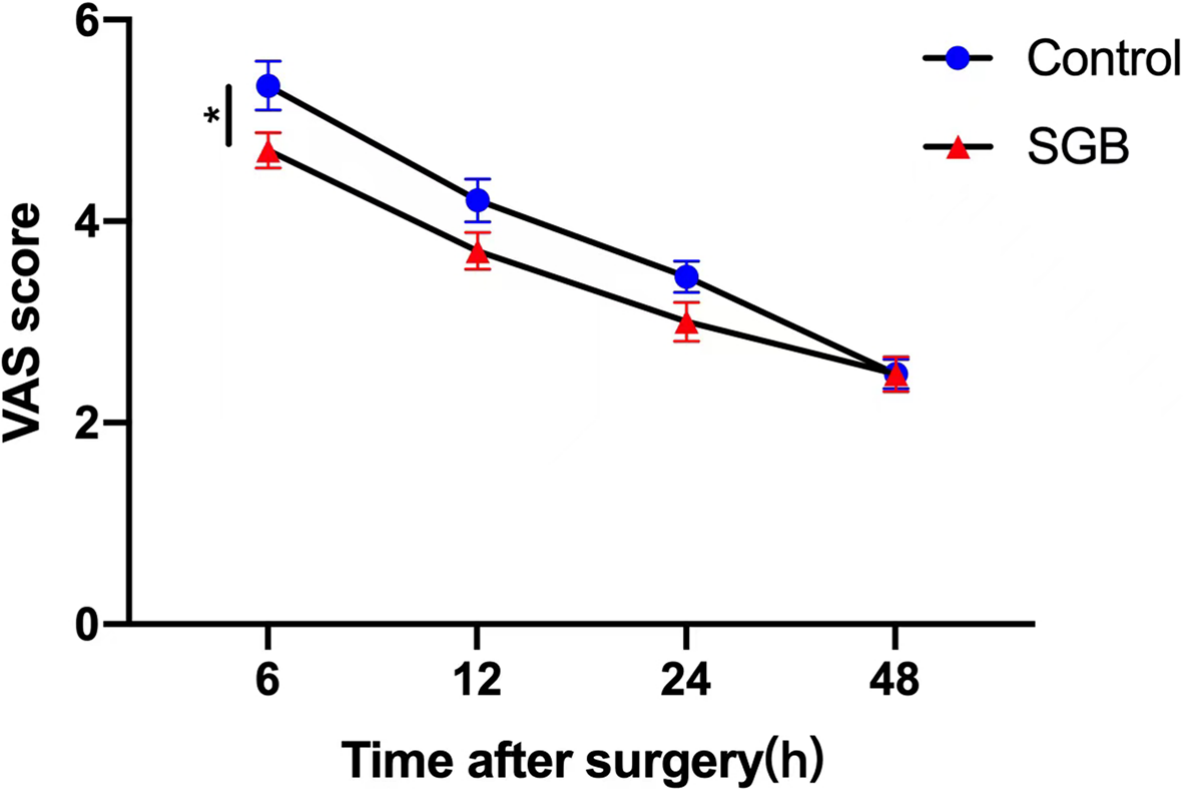
VAS scores at different time point (6 h, 12 h, 24 h, 48 h) after surgery between Control group and SGB group. VAS, Visual Analogue Scale. SGB, stellate ganglion block. Data are presented as mean ± SD. **Means a significant *P* value (*p *< 0.01)

**Fig. 5:**
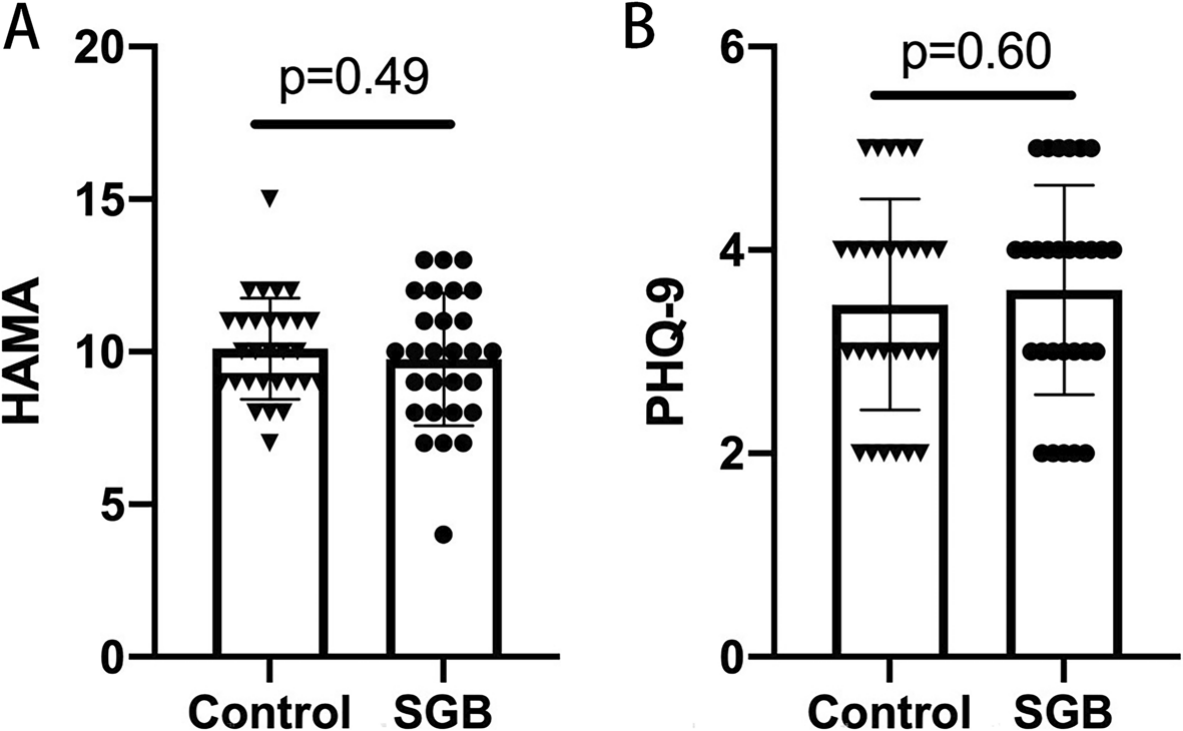
The scores of HAMA and PHQ-9 accessed 3 days after surgery between Control and SGB group. Data are presented as mean ± SD. HAMA, Hamilton Anxiety Scale. PHQ-9, Patient Health Questionnaire-9

Postoperative pain, as measured by VAS, was significantly lower in the SGB group compared to the control group at 6 h post-surgery, with mean [SD] of 4.70 [0.91] and 5.35 [1.32], respectively (*p* = 0.040) (Fig. 4). No significant differences were observed at 12, 24, and 48 h postoperatively, indicating effective pain relief shortly after surgery. Given the approximate 6.8-h average duration of 0.2% ropivacaine's analgesic effect [19], this result further supports the credibility of the analgesic effect. In terms of stress response, both intraoperative and postoperative plasma cortisol concentrations, along with blood glucose levels, were evaluated. The plasma cortisol concentrations in the SGB group were significantly reduced postoperatively (mean [SD], preoperatively, 9.11 [2.37]; postoperatively, 4.01 [3.42], *p* = 0.04) (Fig. 3C). However, cortisol levels in the control group remained largely unchanged (mean [SD], preoperatively, 9.00 [3.48]; postoperatively, 7.75 [3.13]), with a significant difference observed between the two groups (*p* = 0.02) (Fig. 3C). No significant difference was found in blood glucose levels between the two groups (*p* = 0.42, Fig. 3D). Evaluation of anxiety and depression using HAMA and PHQ-9 scores showed comparable results in both the control and SGB groups (Fig. 5). No harm or unintended intervention-related effect occurred in both groups.

### Reduced hospital stays and postoperative morbidity was observed after SGB treatment

Postoperative hospital stays were significantly shorter in the SGB group compared to the control group (*p* = 0.02) (Fig. 3B). Because there were substantial differences in the duration of preoperative examinations among patients admitted to our institution, we conducted additional analysis on postoperative hospital stays. The results demonstrated a significant reduction in average postoperative hospitalization duration in the SGB group, which was 6.61 days, compared to the control group's 8.72 days (*p* = 0.042) (Table 2).

Among the patients, a total of 20 had intraoperative complications (11 in the SGB group, 9 in the control group), with no significant difference between the two groups (*p* = 0.514) (Table 2). Hypotension was a major intraoperative complication. Postoperative complications were experienced by a substantial proportion of patients (2 in the SGB group, 8 in the control group), including nausea, vomiting, and abdominal distension; however, SGB treatment significantly reduced postoperative morbidity (Table 2, *p* = 0.042). Regarding patient cost, both treatment groups exhibited similar number (Table 2). No complications resulted from SGB, and no adverse events related to this study were reported.

Five more patients were lost to follow-up (2 in SGB, 3 in Control) as we failed to contact them after 3 years. Despite this, among the responses we received, 3-year mortality remains similar between the two groups, suggesting that a longer follow-up period may be necessary for detecting potential differences.

## Discussion

This RCT involving patients undergoing elective laparoscopic radical resection of CRC highlights significant benefits associated with preoperative SGB. Notably, it demonstrates improvements in intestinal function, stress levels, and postoperative pain relief immediately after surgery. To assess whether these short-term enhancements translate into meaningful postoperative recovery benefits, we conducted an extended follow-up. Our findings revealed a notable reduction in postoperative morbidity and hospital stay following a single pre-incision SGB procedure. Importantly, these outcomes are in line with the principles of ERAS [5], which is gaining increasing attention in various surgical disciplines, particularly in colorectal surgeries.

Our study contributes to the existing literature by demonstrating that pre-incision SGB significantly accelerates postoperative recovery of bowel function, reduces short-term postoperative pain scores, and lowers postoperative cortisol levels [9, 10]. While previous studies have reported similar findings regarding intestinal function recovery after surgery, our study uniquely evaluates overall patient recovery during the perioperative period using ERAS evaluation criteria. Furthermore, our study confirms a reduction in average hospital stay length and demonstrates decreased postoperative morbidity, including postoperative nausea and vomiting (PONV), hypotension, fever, and bloating, following pre-incision SGB. These findings are novel and warrant further investigation in future studies involving ERAS indicators across various surgical contexts.

Perioperative stress significantly impacts patients' recovery, with the sympathetic nervous system (SNS) playing a crucial role [20]. SGB has been shown to modulate and stabilize the SNS—a mechanism often associated with the treatment of conditions like post-traumatic stress disorder (PTSD) [7]. Our study aligns with previous research by demonstrating a reduction in postoperative plasma cortisol levels following SGB [10]. Additionally, SGB has been found to alleviate stress and inflammatory factors, further supporting its role in promoting rapid postoperative recovery [21–24]. Notably, our study also observed a decrease in norepinephrine (NE) levels following SGB in patients undergoing CRC surgery, raising questions about its potential impact on perioperative cardiovascular complications [10].

Although our study did not reveal significant differences in intraoperative morbidity reported before [15, 25], consistent and skilled operators, along with the use of ultrasound guidance [11, 26], likely contributed to this outcome. Furthermore, our study's design, which involved performing SGB after anesthesia induction, minimized immediate complications. The predominant intraoperative complication observed was hypotension, a common side effect of general anesthesia [27, 28], rather than a direct result of SGB. The occurrence of this complication can be attributed to the general anesthesia itself and patients' individual cardiovascular function.

In our postoperative follow-up, we observed a significant reduction in VAS pain scores at 6 h after surgery following SGB treatment, aligning with previous research. However, no significant differences were found at later time points, possibly due to the short duration of ropivacaine's analgesic effect [29, 30]. Moreover, due to the minimally invasive nature of laparoscopic surgery, discerning pain differences related to wound healing can be challenging. Similar findings were reported in a previous study on the analgesic effect of SGB after upper limb orthopedic surgery, where lower VAS scores were observed only at 4 and 6 h post-surgery [31]. While pre-incision SGB has shown a robust analgesic effect in various models, its efficacy may vary depending on the surgical procedure [32, 33]. For instance, in a randomized controlled study, no significant difference in pain severity was found in open upper extremity surgery after SGB, likely due to the more invasive nature of the procedure [34].

Despite these contributions, our study has limitations. Other indicators such as regional hemodynamics and additional biomarkers of stress responses were not evaluated. Additionally, while our sample size may seem small, it was determined based on previous research [9, 10] and pilot studies. Finally, variations in surgeons' techniques may have influenced our study outcomes, although efforts were made to ensure baseline procedural consistency.

## Conclusions

In summary, the data from this randomized controlled study support the positive effects of SGB in promoting the postoperative recovery of patients undergoing laparoscopic CRC surgery. With further research and exploration, SGB may emerge as a valuable tool in the quest to improve the survival and quality of life for individuals undergoing CRC surgery.

## What is known

1. Colorectal cancer (CRC) is the third most deadly and fourth most commonly diagnosed cancer in the world.
2. The concept of enhanced recovery after surgery (ERAS) has gained more and more attention in various operations, especially in colorectal surgeries.

## What is new

1. Stellate ganglion block (SGB) has been shown to promote the recovery of intestinal function after surgery, which is accompanied by short-term stress relieving
2. Preoperative SGB promotes the postoperative recovery of patients undergoing laparoscopic CRC surgery.

## Data Availability

The datasets used and analysed during the current study available from the corresponding author on reasonable request.
